# Co-Rooming Accounts for Socioeconomic Disparities in Infant Sleep Quality among Families Living in Urban Environments

**DOI:** 10.3390/children9101429

**Published:** 2022-09-21

**Authors:** Morgan A. Finkel, Sonya V. Troller-Renfree, Jerrold S. Meyer, Kimberly G. Noble

**Affiliations:** 1 Division of Child and Adolescent Health, Department of Pediatrics, Columbia University Irving Medical Center, 622 West 168th St., VC 417, New York, NY 10032, USA; 2 Department of Biobehavioral Sciences, Teachers College, Columbia University, 525 West 120th St., Box 199, New York, NY 10027, USA; 3 Department of Psychological and Brain Sciences, University of Massachusetts Amherst, Tobin Hall, Amherst, MA 01003, USA

**Keywords:** infants, sleep, socioeconomic disparities, infant co-rooming, maternal stress

## Abstract

Poor infant sleep quality is associated with negative maternal and infant health outcomes. This study measures socioeconomic disparities in infant sleep quality, and assesses whether child sleep location and maternal stress mediate associations between socioeconomic status (SES) and infant sleep quality. The study includes 86 socioeconomically diverse, mother-infant dyads living in an urban area with infants aged 6–12 months. Mothers reported socioeconomic demographics, infant sleep quality (Brief Infant Sleep Questionnaire) and maternal subjective stress (Perceived Stress Scale). Maternal objective stress was measured via hair cortisol concentration (HCC). The associations among SES, infant sleep quality, infant co-rooming, and maternal stress were assessed. Infants from families with lower income-to-needs (ITN) ratios had poorer infant sleep quality. The association between familial ITN and infant sleep quality was mediated by whether the child co-rooms with parents. Maternal perceived stress was independently associated with infant sleep quality, but HCC was not associated with infant sleep quality.

## 1. Introduction

Sleep disturbances occur in 10–36% of infants, and many of these problems persist into later childhood [1,2]. Poor infant sleep can cause significant parental distress and is associated with increased rates of postpartum depression and poor maternal health [3]. Research has also linked infant sleep problems with a variety of poor health and developmental outcomes in children. Poor sleep consolidation (i.e., failure to establish uninterrupted sleep during the night) and later bedtimes are associated with worse neurodevelopmental and social-emotional outcomes [4,5]. In addition, severe and persistent sleep issues during infancy increase the odds of subsequent anxiety and emotional disorders [6].

Socioeconomic disparities in childhood sleep quality have been well documented in older children, and recent research suggests that these disparities emerge during infancy [7,8,9]. Given the association between sleep and subsequent health and developmental outcomes, it has been proposed that differences in sleep quality may mediate or moderate socioeconomic disparities in child health and development [10]. For this reason, improving infant sleep quality may be an important target to decrease health and developmental disparities in children. However, the literature lacks studies investigating the mechanisms underlying socioeconomic disparities in infant sleep quality.

We hypothesized that familial income and parental education would be associated with infant sleep quality. In addition, we hypothesized that, among infant-mother dyads living in urban settings, room sharing and parental stress would play important mechanistic roles in the relationship between familial socioeconomic status (SES) and infant sleep quality. Urbanicity is a known risk factor for poor infant sleep, suggesting that infants in urban environments face unique barriers to quality sleep [11]. Infant-parent room sharing is associated with poorer infant sleep consolidation, and household crowding, which is more commonly found in urban, socioeconomically disadvantaged settings, may lead to increased rates of obligatory room sharing [12,13]. Studies have also linked poor child sleep to increased levels of parental stress, and stressful life events occur more frequently in socioeconomically disadvantaged families [14,15]. Chronic stress in adults has been linked to sleep disturbances, and a study of women’s sleep found that perceived stress mediated the relationship between income and sleep disturbances [16,17]. 

In order to lay the groundwork for interventions that mitigate socioeconomic disparities in infant sleep quality in urban settings, this study aims to (1) assess socioeconomic disparities in infant sleep quality in a large urban city, (2) determine whether child sleep location in the home mediates the relationship between SES and infant sleep quality, and (3) determine whether perceived and/or physiologic measures of maternal stress mediate the relationship between SES and infant sleep quality.

## 2. Methods

### 2.1. Study Population and Setting

Participants were recruited from the New York metropolitan area as part of a cross-sectional study investigating the association between early life experiences and infant development. A cohort of 94 socioeconomically diverse mother-infant dyads was recruited to participate when their infants were 6, 9, or 12 months of age. Inclusionary criteria included infant age between 5.5 and 12.5 months and infants born at or after 36 weeks gestation without neurological or developmental complications. Siblings and multiples were excluded. The convenience sample of mothers was recruited via flyers in the community and WIC clinics, postings on the laboratory website, and tabling at community events. Mothers were required to be fluent in either English or Spanish. Study protocol was approved by the Institutional Review Board at Columbia University Teachers College. 

### 2.2. Demographics and SES

Recruited mothers filled out electronic questionnaires in either English or Spanish on parent and child demographics, family size, family annual income, and years of parental education. Familial income was standardized by calculating an income-to-needs ratio (ITN). ITNs were calculated by dividing the total annual familial income by the United States federal poverty threshold for the specified family size for the specified year. The United States federal poverty threshold is set at three times the cost of a minimum food diet in 1963, adjusted for family size and for inflation [18], and does not vary by location across the United States. The 2022 federal poverty threshold for a family of 4 was set at USD 27,750; therefore, in 2022, a family of 4 with a gross income of USD 27,750 would have an ITN ratio of 1. The federal poverty threshold is used for determining eligibility for certain government run programs. For example, for most families with children, the eligibility threshold for New York State supplemental nutrition program is 200% of the federal poverty threshold [19]. As ITN ratios were expectedly positively skewed in our sample, ratios were log transformed prior to analysis. Values more than three standard deviations from the mean were winsorized. Average parental education was calculated as a mean of maternal and paternal years of education. Alternatively, maternal education was used alone when paternal education was not reported (*n* = 16). Reported models utilize the average parental education variable, but results were similar in magnitude when maternal education was used instead. 

### 2.3. Infant Sleep Measures

Infant sleep was measured using the original Brief Infant Sleep Questionnaire (BISQ) [20]. 86 of the 94 participants filled out the BISQ (eight chose not to complete this questionnaire). The questionnaire contains ten items that measure infant sleep patterns and environment, including a question about child sleep location. BISQ measures have been shown to correlate significantly with sleep patterns measured by actigraphy and sleep diaries, and responses have high test–retest reliability (r > 0.82) [20]. 

#### 2.3.1. Infant Sleep Quality

The main infant sleep outcome of interest in this study is infant sleep quality, as measured by the modified Infant Sleep Subscale (mISS). Recent research on the revised version of the BISQ established a validated age-normalized scoring system, employing five questions to calculate an Infant Sleep Subscale [21]: (1) frequency of nighttime wakening; (2) length of time to put baby to sleep; (3) time child spends sleeping at night; (4) time child spends awake at night; and (5) the longest stretch of time that child is asleep during the night without waking up. As described in more detail in the validation study, scoring of the items in the subscale are derived from age-based normative U.S. data with each question scored on a scale of 0–100 with higher scores representing better sleep quality [21]. The subscale score is calculated using a weighted average based on rankings from independent sleep specialists on the importance of each item. The original BISQ used in the present study contains the first four of the Infant Sleep Subscale items. In consultation with the BISQ creators, we calculated a modified Infant Sleep Subscale (mISS) for each participant, with a missing or null value for the missing 5th question in the function (E-mail communication with study@babysleep.com on 12 August 2021). Age-referenced mISS scores range from 0–100, with higher scores representing more desired sleep patterns.

#### 2.3.2. Dichotomized Child Sleep Location 

The original BISQ asks parents to report their child’s sleeping arrangement. The options are: infant crib in a separate room, infant crib in parents’ room, in parents’ bed, infant crib with sibling, or other (asks to specify). For analysis purposes, responses were collapsed into two categories: infants who room with their parents (infants who sleep in a crib in their parents’ room or sleep in their parents’ bed) and infants who sleep in a different room (infants who sleep in a crib in a separate room or in a crib with a sibling or other relative). Parents who responded “other” were categorized based on their free text responses. We chose to dichotomize this variable because mISS scores for infants who slept in their parents’ bed (59.1 ± 14.4, *n* = 19) and mISS scores for infants who slept in a crib in their parents’ room (64.6 ± 21.1, *n* = 42) were not significantly different (*p* = 0.32), and only 4 infants were reported to sleep with a sibling or other family member besides their parents. 

#### 2.3.3. Dichotomized Infant Clinical Sleep Problem

A secondary sleep measure assessed was whether the infant met criteria for having a clinical sleep problem. This was defined as meeting at least one of the following clinical cut-offs: (1) child wakes >3 times per night; (2) nocturnal wakefulness is >1 h; or (3) total sleep time is <9 h. These cut-offs were described in the initial BISQ validation study [20].

### 2.4. Maternal Perceived Stress 

Maternal perceived stress was measured with the Perceived Stress Scale (PSS) [22,23]. The PSS is a validated 10-item questionnaire that asks respondents to rate on a 5-point scale (0–4) how often they have felt stressed about different aspects of their life in the past month. PSS scores correlate with a variety of health measures, including depressive and physical symptomatology [22]. If participants were missing ≤10% of the 10 items (1 item missing, *n* = 3), missing items were mean-replaced with responses of the other items to calculate the total score which ranges from 0–40. PSS scores from 0–13 are considered low perceived stress, 14–26 moderate perceived stress, and 27–40 high perceived stress. 

### 2.5. Maternal Physiologic Stress

Maternal physiologic stress was measured via hair cortisol concentration (HCC), which reflects circulating cortisol levels during the period of hair growth [24]. HCC is a relatively new, non-invasive tool for measuring physiologic chronic stress, and is elevated in groups with ongoing stressors, but not consistently associated with self-reported measures of perceived stress [25]. Research staff cut a small section of hair near the posterior vertex of the mother’s scalp. Each sample weighed at least 15 mg and was trimmed to be 3 cm from the scalp to the end. Since hair grows about 1 cm per month on average, the 3 cm sample reflects circulating cortisol levels over the ~3 months prior to sampling. Hair samples were stored at −20° Celsius before being sent to a lab at the University of Massachusetts for processing as previously described [26]. 65 out of 94 mothers had hair samples that were collected and fully processed. The remaining mothers were missing hair samples because they preferred not to provide a sample (*n* = 10); their hair was too short or inaccessible due to styling (*n* = 11); a different caregiver attended the lab visit (*n* = 3); or the hair sample was inadequate (*n* = 5). Of the 65 samples collected, 9 were discarded because mothers reported use of systemic or topical corticosteroids, which can affect HCC. In addition, one sample with a concentration outside the physiological range (>100) was discarded, leaving 55 participants with usable HCC results. Of these, 51 participants also had BISQ results, constituting our final sample in HCC analyses. As HCC was positively skewed, values were log transformed prior to analysis. There were no significant associations between HCC and frequency of hair washing, use of oral contraceptives, or use of hair dye. 

### 2.6. Statistical Analysis 

Univariate linear regressions were run to determine appropriate covariates to include in models. None of the potential demographic covariates, including child sex, ethnicity and race, parental age, or number of children in the home were significantly associated with the mISS, and therefore none were included as control variables in final models. Infant age was not included in models as mISS scores are age-referenced. 

Separate univariate linear regressions were run to test the hypotheses that ITN and parental education were associated with mISS. Univariate regressions were also run to test whether potential mediators (perceived maternal stress and child sleep location) were associated with the mISS. Separate mediation models were then used to test whether any relationships between measures of SES (ITN and parental education) and infant sleep quality (mISS) were mediated by child sleep location (infants who room with their parents versus infants who sleep in a different room) and perceived maternal stress (PSS). 

A similar analysis was performed on data from the subsample of 51 participants from whom we had HCC, in order to test the hypothesis that any links between SES (ITN, parental education) and infant sleep quality (mISS) are mediated by maternal physiological stress (maternal HCC). 

Lastly, a secondary post hoc analysis was performed using logistic and firth logistic regressions to see if the same measures of SES, maternal stress, and sleep location increased the odds of the child meeting criteria for a clinical sleep problem. Child age was included as a control variable in these models. 

SPSS Statistics Version 25 was used for linear and logistic regression models. SAS Version 9.4 was used to test mediation models and run firth logistic regressions when regular logistic regression was not appropriate. The dataset analyzed during the current study is not publicly available, but is available from the corresponding author on reasonable request.

## 3. Results

### 3.1. Demographics 

86 mother-infant dyads were included in the analysis. The three age groups (6 months, 9 months, and 12 months) were approximately evenly distributed in the sample (Table 1). Sixty-six percent of the children were male. The children represented a racially, ethnically, and socioeconomically diverse group. About a quarter reported that their child is Black, and about 45% reported that their child is Hispanic. The median ITN ratio was 2.3; 26 participants (31%) had an ITN < 1 and 40 participants (48%) had an ITN < 2. Average parental educational attainment was 15.2 years. mISS scores ranged from 6.2 to 97.9 with a mean score of 67.6 ± 19.9. Average PSS score fell in the moderate stress category (20.6 ± 7.9).

### 3.2. Univariate Analyses of SES, Child Sleep Location, Maternal Stress, and Infant Sleep Quality (mISS) 

In univariate analysis, children from families with lower ITN ratios were at higher risk for poorer infant sleep quality (β = 7.69, *p* = 0.033)**.** Parental education was marginally related to infant sleep quality with infants whose parents had less education at borderline higher risk for poorer infant sleep quality (β = 1.06, *p* = 0.070). Infants who slept in a room with their parents had significantly worse sleep quality when compared to infants who slept in a room without their parents (β = −16.34, *p* < 0.001). Mothers who reported higher perceived stress (PSS) had infants with worse sleep quality (β = −0.87, *p* = 0.001). Finally, in the subgroup analysis of infants with usable maternal HCC, maternal HCC was not associated with infant sleep quality (β = −1.69, *p* = 0.43). 

### 3.3. Investigating Child Sleep Location as a Potential Mediator of the Link between SES and Infant Sleep Quality (mISS)

**Figure 1:**
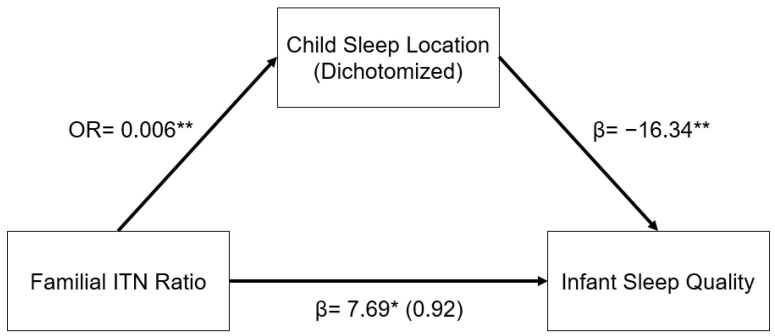
Regression coefficients or odds ratio for the relationship between familial income-to-needs (ITN) ratio and infant sleep quality (mISS) as mediated by child sleep location (1 = room with parents). Child sleep location is a significant indirect mediator of the link between familial ITN and infant sleep quality. The regression coefficient between familial ITN ratios and mISS, controlling for child sleep location, is in parentheses. ** *p* < 0.01, * *p* < 0.05.

The relationship between familial ITN ratios and infant sleep quality was mediated by child sleep location. As illustrated in the indirect pathway in Figure 1, in a logistic regression model, higher familial ITN was associated with decreased odds of having an infant who slept in a room with their parents as opposed to in a room without their parents (OR: 0.006, 95% CI: 0.001–0.070, *p* < 0.001). In addition, infants who slept in a room with their parents had significantly lower infant sleep quality (β = −16.34, 95% CI: −25.11–−7.57, *p* < 0.001). This indirect pathway from familial ITN to infant sleep quality through child sleep location was significant (β = 6.77, 95% CI: 1.53–12.01, *p* = 0.011). In other words, infants from higher-income homes were less likely to sleep in a room with their parents, and infants who sleep in a room with their parents had worse sleep quality, as reported by their mothers.

**Figure 2:**
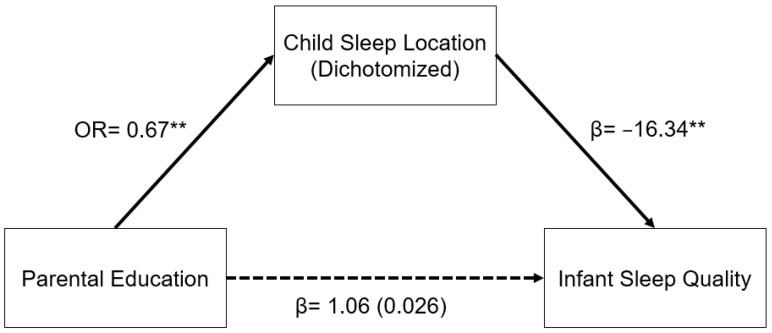
Regression coefficients or odds ratios for the relationship between years of parental education and infant sleep quality (mISS) as mediated by sleep location (1 = room with parents). Child sleep location is a significant indirect mediator of the link between parental education and infant sleep quality. The regression coefficient between parental education and mISS, controlling for sleep location, is in parentheses. ** *p* < 0.01.

A similar relationship was seen in the model for parental education (Figure 2). In a logistic regression model, increased parental education was associated with decreased odds of having an infant who slept in a room with their parents as opposed to in a room without their parents (OR: 0.67, 95% CI: 0.55–0.81, *p* < 0.001). Sleep location and infant sleep quality were related as described above. The indirect pathway from parental education to infant sleep quality through child sleep location was significant (β = 1.03, 95% CI: 0.30–1.76, *p* = 0.006). 

### 3.4. Comparing Measures of SES and Family Size by Child Sleep Location 

Infants who slept in a room with their parents has significantly lower familial ITN ratios (ITN = 1.1 ± 1.6) as compared to infants who slept in a room without their parents (ITN = 2.2 ± 1.3, t(82) = 6.7, *p* < 0.001). Infants who slept in a room with their parents also had parents with significantly fewer years of education (14.0 ± 3.4 years) as compared to infants who slept in a room without their parents (18.1 ± 2.5 years, t(84) = 5.5, *p* < 0.001). Family size was marginally associated with child sleep location. Infants who slept in a room with their parents had on average larger families (4.1 ± 1.7 people) as compared to infants who slept in a room without their parents (3.5 ± 0.6 people, t(83) = −1.7, *p* = 0.099).

### 3.5. Investigating Perceived Maternal Stress as a Potential Mediator of the Link between SES and Infant Sleep Quality (mISS)

The relationship between familial ITN ratios and infant sleep quality was not directly mediated by perceived maternal stress (PSS), as familial ITN ratios and perceived maternal stress scores were not significantly related (β = −0.85, 95% CI: −3.78–2.09, *p* = 0.57). However, higher perceived maternal stress was significantly associated with reduced infant sleep quality (β = −0.87, 95% CI: −1.39–0.35, *p* = 0.001). In a model including both familial ITN and perceived maternal stress, both were significantly associated with infant sleep quality (Familial ITN: β = 8.44, 95% CI: 1.63–15.25, *p* = 0.016; PSS: β = −0.83, 95% CI: −1.34–−0.33, *p* = 0.002). 

A similar relationship was seen in the model for parental education. The association between parental education and infant sleep quality was not significantly mediated by perceived maternal stress. Parental education and perceived maternal stress were not significantly related (β = 0.053, 95% CI: −0.40–0.50, *p* = 0.82). However, higher perceived maternal stress was significantly associated with worse infant sleep quality as described above. In a model including both parental education and perceived maternal stress, higher parental education was associated with significantly better infant sleep quality, and higher perceived maternal stress was associated with significantly poorer infant sleep quality (parental education: β = 1.21, 95% CI: 0.13–2.29, *p* = 0.028; PSS: β = −0.89, 95% CI: −1.40–−0.39, *p* < 0.001). 

### 3.6. Investigating Physiologic Maternal Stress as a Potential Mediator of the Link between SES and Infant Sleep Quality (mISS)

A separate sub-group analysis was performed on the data from the 51 participants who had useable maternal HCC. Maternal HCC was not significantly related to infant sleep quality (β = −1.69, 95% CI: −5.96–2.58, *p* = 0.43). Higher familial ITN was associated with significantly lower maternal HCC, but average parental education was not (familial ITN: β = −0.62, 95% CI: −1.2–−0.024, *p* = 0.042, parental education: β = −0.051, 95% CI: −0.14–0.039, *p* = 0.26). In this subgroup, familial ITN and parental education were both marginally associated with infant sleep quality (familial ITN: β = 6.93, 95% CI: −0.25–14.12, *p* = 0.058, parental education: β = 0.99, 95% CI: −0.16–2.13, *p* = 0.091). Maternal HCC was not a significant mediator of either of these relationships. In addition, parental perceived stress (PSS) was not significantly associated with maternal HCC (β = −0.006, 95% CI: −0.053–0.040, *p* = 0.79).

### 3.7. Multivariable Assessments of SES, Child Sleep Location, Perceived Maternal Stress, and Infant Sleep Quality (mISS)

In final models including familial ITN and perceived maternal stress, both were significantly associated with infant sleep quality (Table 2, Model 1). When the mediator, child sleep location, was added to the model, ITN was no longer significant, but the association between perceived maternal stress and infant sleep quality remained relatively unchanged and significant (Table 2, Model 2). A similar pattern was seen for the analogous models using parental education instead of ITN (Table 2, Models 3 and 4). 

### 3.8. Risks for Infant Clinical Sleep Problem 

In a secondary post hoc analysis we assessed whether the measures of SES, maternal stress and child sleep location was related to the odds of a child meeting criteria for a clinical sleep problem. Using logistic regression, we found that, when controlling for infant age, both increased familial ITN and increased parental education were associated with decreased odds of having an infant meeting criterion for a clinical sleep problem (familial ITN OR: 0.40, 95% CI: 0.17–0.95, *p* = 0.037; parental education OR: 0.85, 95% CI: 0.73–0.99, *p* = 0.037). Parental perceived stress was marginally related to higher odds of an infant sleep problem (OR: 1.07, 95% CI: 1.0–1.15, *p* = 0.059) and, in the subsample of 51 families, maternal HCC was significantly related to higher odds of an infant clinical sleep problem (OR: 1.9, 95% CI: 1.1–3.4, *p* = 0.025). Using firth logistic regression, we found that infants who slept in a room with their parents had 24.8 times the odds of meeting criteria for a sleep problem as compared to infants who did not sleep in a room with their parents (OR = 24.8, 95% CI: 1.4–443.7, *p* = 0.029).

## 4. Discussion

In this study of mother-infant dyads living in an urban setting, we hypothesized that family SES (familial income and parental education) would be directly related to infant sleep quality, and that the relationship between SES and infant sleep quality would be mediated by child sleep location, as well as by maternal perceived and physiologic stress. Consistent with prior studies [7,8,9], we found that higher family income was indeed significantly associated with better infant sleep quality. This association was largely accounted for by infant co-rooming practices, and not maternal stress. These results are consistent with prior studies that find that co-rooming is associated with more difficulty consolidating overnight sleep [12]. We believe that this association is likely due to increased parental interventions and less self-soothing behaviors when the child wakes overnight. However, it is also possible that infants have similar sleep quality, but that mothers are less aware of sleep disruptions when infants are sleeping in a different room.

For many lower-income families, co-rooming is a necessity rather than a choice, given heightened space restrictions in crowded urban environments [13,27]. The new American Academy of Pediatrics Policy Statement on Sleep-Related Infant Deaths recommends “that infants sleep in the parents’ room, close to the parents’ bed, but on a separate surface designed for infants, ideally for at least the first 6 months,” in order to decrease the risk of infant sleep-related death [28]. The AAP report cites the significant increased risk of sudden death for infants that sleep in a separate room, as opposed to room sharing without bed sharing. However, they state that “there is no specific evidence for when it might be safe to move an infant to a separate room before 1 year of age.” Given our findings that co-rooming likely contributes to worse infant sleep quality in a population of infants aged 6 to 12 months, future research examining the safety of separate room sleeping in the second half of the infant’s first year is needed. In addition, future studies should delve deeper into the co-rooming sleep environment to understand how sleep arrangement, lighting, noise, temperature, and presence of electronics contribute to sleep quality for both infants and parents, with the goal of adjusting the co-rooming environment to make it more sleep-friendly for infants and parents. 

While maternal perceived stress did not mediate the association between SES and infant sleep quality, it was separately associated with infant sleep quality, independent of familial SES. Mothers who reported higher levels of stress had infants with lower sleep quality. SES was not significantly related to perceived stress, perhaps because parents of young infants across the socioeconomic spectrum face many similar stressors. The relationship between parental stress and infant sleep is likely bidirectional, with parental stress poorly impacting infant sleep consolidation through altered parenting practices and household chaos [29], and poor infant sleep leading to increased maternal stress [30]. This maternal stress-infant sleep relationship likely plays a role in the increased risk of postpartum depression seen in mothers of infants with sleep problems [31]. It is also possible that one or more additional variables, such as maternal symptoms of anxiety or depression, may account for the significant relationship between maternal perceived stress and parentally reported sleep quality. Other studies have documented the relationship between perceived stress and maternal mental health [32,33], and mothers with worse mental health may have a tendency to report poorer sleep for their infants. Future studies that measure parental mental health and objective measures of infant sleep are required to clarify this relationship.

In contrast to perceived stress, lower family income was associated with higher maternal physiologic stress, as operationalized by maternal HCC, as has been previously described [34]. Prior work has also suggested that the correlation between perceived and physiologic stress is inconsistent [25]. Contrary to hypotheses, we did not find that higher maternal HCC was associated with worse infant sleep quality, and maternal HCC did not mediate the relationship between familial income and infant sleep quality. However, in post hoc analyses, infants of mothers with higher HCC had significantly higher odds of having infants that met clinical criteria for a sleep problem. Previous research in the present cohort of infants reported that infants of mothers with elevated physiologic stress had alterations in brain function as compared to infants of mothers with lower physiologic stress [35]. It is possible that these differences in brain development affect sleep consolidation and increase the odds of having a clinical sleep problem. It is also possible that mothers of infants who have poor sleep consolidation have elevated physiologic stress from their own lack of sleep. Future studies should use larger sample sizes and assess both maternal and infant HCC and sleep patterns.

Our study has notable strengths. The sample included a diverse group of participants with a wide range of familial income and parental education. We used age-reference standardized scoring of the BISQ which has been studied extensively on a diverse population of infants. In addition, by focusing on a population of families living in an urban setting, our results are clinically relevant to infants living in cities who are at higher risk for sleep problems. Finally, we investigated several potential mediating pathways, measuring both perceived and physiologic stress. 

As with any study, there are also limitations. This study is cross-sectional and correlational, and therefore directionality and causality of relationships are not clear. The participants were recruited from a convenience sample and therefore are not reflective of the city’s population as whole. In addition, we relied on parental report of infant sleep as opposed to more objective measures such as actigraphy. The study also had a relatively small sample size, particularly for the sub-analysis of maternal HCC. This limited us from analyzing each sleep location arrangement separately as opposed to in a dichotomized fashion. We also did not know if other family members outside of the parents and participant child were sleeping in the parents’ room. Finally, given that we used an older version of the BISQ, we had a missing question when calculating the infant sleep subscale, requiring us to create a modified infant sleep subscale. Future studies assessing socioeconomic disparities in infant sleep should be larger, longitudinal, and consider delving deeper into the home sleeping environment and maternal sleep.

## 5. Conclusions

In a group of infants living in an urban environment, we found that socioeconomic disparities in infant sleep quality are most prominently associated with family income. This disparity seems to be almost completely mediated by differences in sleep arrangements, with infants from more impoverished homes more likely to co-room with their infants, and in turn, infants who co-room at higher risk for worse sleep quality. Parental perceived stress was separately associated with infant sleep quality, with infants from high-stress homes at higher risk for poor sleep. Maternal physiologic stress did not mediate the relationship between income and our continuous infant sleep quality measure, but may be related to the risk of infants developing clinically significant sleep problems. Given these inconsistent results, further research is required to clarify the relationship between maternal physiological stress and infant sleep. Interventions aimed at improving infant sleep quality for populations in urban settings should recognize the potential impacts of perceived maternal stress on infant sleep, and consider the challenges of co-rooming for low-income families. 

## Figures and Tables

**Table 1:** Demographic Characteristics of study sample (*n* = 86).

Variable		Frequencies or Descriptives	
Child Characteristics			
Age: N (%)	6 months 9 months 12 months	29 29 28	(33.7) (33.7) (32.6)
Sex: N (%)	Female Male	29 57	(33.7) (66.3)
Child Race: N (%)	White Black Asian American Indian/Native Alaskan Other Prefer not to answer	27 21 1 1 24 12	(31.4) (24.4) (1.2) (1.2) (27.9) (14.0)
Child Ethnicity: N (%)	Hispanic Not Hispanic Prefer not to answer	39 40 7	(45.3) (46.5) (8.1)
Child Sleep Location: N (%)	Room with Parents Different room	61 25	(70.9) (29.1)
Gestational age (weeks, *n* = 81): Mean (SD)		39.6	(1.2)
Family and Parental Characteristics			
Familial ITN ^1^ Ratio (*n* = 84): Median (IQR)		2.3	(0.7–5.6)
Annual Familial Income (USD 1000 s, *n* = 84): Median (IQR)		50	(20–125)
Family Size (*n* = 85): Mean (SD)		3.9	(1.5)
Children Supported (*n* = 85): Median (IQR)		1.0	(1.0–2.0)
Average Parental Education (years, *n* = 86): Mean (SD)		15.2	(3.7)
Sleep and Stress Measures			
Modified Infant Sleep Scale (*n* = 86): Mean (SD)		67.6	(19.9)
Infant Meeting Criteria for a Clinical Sleep Problem ^2^: N (%)	Yes No	19 67	(22.1) (77.9)
Perceived Stress Scale (*n* = 84): Mean (SD)		20.6	(7.9)
Maternal Hair Cortisol (pg/mg, *n* = 51): Median (IQR)		3.4	(1.7–6.4)

^1^ ITN = annual familial income divided by the family size specific federal poverty threshold for the specific year. ^2^ Infant meeting at least one of the following clinical cut-offs: (1) child wakes >3 times per night; (2) nocturnal wakefulness is >1 h; or (3) total sleep time is <9 h.

**Table 2:** Multivariate Linear Regression Results Predicting Modified Infant Sleep Quality Scores.

Model	Unstandardized β (95% CI)	*p*-Value
Model 1		
Income-to-Needs Ratio Perceived Maternal Stress Scale (PSS)	8.44 (1.63–15.25) −0.83 (−1.34–−0.33)	0.016 * 0.002 **
Model 2		
Income-to-Needs Ratio Perceived Maternal Stress Scale (PSS) Child Sleep Location (1 = room with parents)	2.78 (−5.43–10.99) −0.76 (−1.26–−0.27) −12.49 (−23.18–−1.80)	0.50 0.003 ** 0.023 *
Model 3		
Mean Parental Education (years) Perceived Maternal Stress Scale (PSS)	1.21 (0.13–2.29) −0.89 (−1.40–−0.39)	0.028 * <0.001 **
Model 4		
Mean Parental Education (years) Perceived Maternal Stress Scale (PSS) Child Sleep Location (1 = room with parents)	0.33 (−0.88–1.55) −0.77 (−1.26–−0.27) −13.73 (−23.69–−3.78)	0.59 0.003 ** 0.007 **

* *p* < 0.05, ** *p* < 0.01.

## Data Availability

The data presented in this study are available on request from the corresponding author.

## Abbreviations

SES (socioeconomic status), HCC (hair cortisol concentration), ITN (income-to-needs), BISQ (Brief Infant Sleep Questionnaire), mISS (modified Infant Sleep Subscale), PSS (Perceived Stress Scale).
